# Timely Interventions for Children with ADHD through Web-Based Monitoring Algorithms

**DOI:** 10.3390/diseases7010020

**Published:** 2019-02-07

**Authors:** Julia Oppenheimer, Oluwafemi Ojo, Annalee Antonetty, Madeline Chiujdea, Stephanie Garcia, Sarah Weas, Tobias Loddenkemper, Eric Fleegler, Eugenia Chan

**Affiliations:** 1 Department of Neurology, Boston Children’s Hospital, Boston, MA 02115, USA; julia.oppenheimer@umassmed.edu (J.O.); william.femi@gmail.com (O.O.); annalee.antonetty@childrens.harvard.edu (A.A.); madeline.chiujdea@childrens.harvard.edu (M.C.); sgarcia7@pride.hofstra.edu (S.G.); tobias.loddenkemper@childrens.harvard.edu (T.L.); 2 Division of Developmental Medicine, Boston Children’s Hospital, Boston, MA 02115, USA; sarah.weas@childrens.harvard.edu; 3 Division of Emergency Medicine, Boston Children’s Hospital, Boston, MA 02115, USA; eric.fleegler@childrens.harvard.edu; 4 Harvard Medical School, Boston, MA 02115, USA

**Keywords:** trigger, algorithm, alerts, ADHD, Vanderbilt rating scale, parent reports, care plan

## Abstract

The aim of this study was to evaluate an automated trigger algorithm designed to detect potentially adverse events in children with Attention-Deficit/Hyperactivity Disorder (ADHD), who were monitored remotely between visits. We embedded a trigger algorithm derived from parent-reported ADHD rating scales within an electronic patient monitoring system. We categorized clinicians’ alert resolution outcomes and compared Vanderbilt ADHD rating scale scores between patients who did or did not have triggered alerts. A total of 146 out of 1738 parent reports (8%) triggered alerts for 98 patients. One hundred and eleven alerts (76%) required immediate clinician review. Nurses successfully contacted parents for 68 (61%) of actionable alerts; 46% (31/68) led to a change in care plan prior to the next scheduled appointment. Compared to patients without alerts, patients with alerts demonstrated worsened ADHD severity (β = 5.8, 95% CI: 3.5–8.1 [*p* < 0.001] within 90 days prior to an alert. The trigger algorithm facilitated timely changes in the care plan in between face-to-face visits.

## 1. Introduction

In response to the Institute of Medicine’s (IOM) 2001 report on designing a health-care system for the 21st century [1], health care models should encourage interventions that engage patients and families, facilitate early detection of threats to patient safety, and reduce delays in care. Such interventions often leverage health information technology (HIT) to facilitate efficient communication about symptoms, functioning, and response to treatment, from patients and caregivers to clinicians outside of in-person encounters, to promote continuous care [1,2]. 

This is particularly pertinent for children with Attention-Deficit/Hyperactivity Disorder (ADHD), one of the most common neurodevelopmental conditions in the United States. Clinicians who treat pediatric patients with ADHD must rely on information provided by parents, other caregivers, and teachers to continuously monitor a child’s symptoms, functional status, response to medication treatment, and medication side effects [2]. Timely response from clinicians to such parent- and teacher-reported information is essential for improving patient safety and outcomes, and maximizing treatment effectiveness [1,3,4]. However, despite recommendations to use rating scales for monitoring children with ADHD [5] (routine monitoring), clinicians seldom obtain ADHD rating scales at in-person clinical visits, let alone monitor children’s symptoms and functioning in between visits (i.e., remote monitoring) [5].

Digital health technology is an important lever for improving quality of care, while reducing costs for all individuals with chronic conditions, including ADHD [6]. HIT solutions that facilitate remote monitoring have the potential to improve family engagement, patient functioning, and quality of care for children with ADHD [7,8,9,10]. Children with ADHD, caregivers, and health care clinicians have indicated interest in patient-centered, mobile or web-based applications that improve ADHD self-management [9]. Implementation of a web-based ADHD portal designed to administer and score ADHD rating scales can lead to improved quality of care and patient outcomes in a community-based setting, but few such systems are widely available for caregiver use [8].

The volume of generated patient data may overwhelm and reduce the responsiveness of clinicians outside of scheduled office visits [11,12,13,14,15]. Delays in responding to potentially actionable data can lead to otherwise preventable adverse events. Both the IOM and the Institute for Healthcare Improvement (IHI) have called for error-proofing of healthcare delivery systems to ensure early identification of potential adverse events [16,17,18]. Electronic health record (EHR)-based trigger tools have been successful in identifying potential risk of patient harm and improving intervention timeliness [1,19,20,21,22,23,24]. However, little is known about trigger tools embedded within patient- or family-reported outcome systems.

To address these issues, we developed and implemented a web-based patient monitoring platform, TriVox Health, which allows caregivers to report information remotely through regularly-scheduled, disease-specific questionnaires, between clinical visits. Algorithms were programmed to trigger alerts associated with worsened symptoms and side effects. We hypothesized that the trigger algorithms would prompt timely medical decision-making by clinicians, between patient visits, for children with ADHD.

## 2. Materials and Methods

### 2.1. Overall Design

This study was part of a larger hybrid implementation-effectiveness cluster randomized clinical trial evaluating the impact of TriVox Health on care delivery and patient outcomes (clinicaltrials.gov NCT02097355). Primary caregivers registered in TriVox Health for routine clinical care provided informed consent to participate in the optional research study, which consisted of additional periodic surveys and review of medical records. For this analysis, the intervention of interest was a trigger algorithm embedded within the TriVox Health ADHD questionnaire used for routine ADHD care in the Department of Neurology at Boston Children’s Hospital. We collected data at point-of-care events and reviewed additional pertinent information from patient medical records. The Boston Children’s Hospital Institutional Review Board approved this study.

### 2.2. Participants and Setting

In 2014, the Department of Neurology’s five outpatient clinic locations and one hundred and thirteen clinicians provided care for nearly five thousand children with ADHD. Participation in TriVox Health for routine ADHD care was voluntary for Neurology clinicians and for parents. For this study, we limited the analysis to parents of patients who received ongoing care for ADHD and were prescribed ADHD medications. Parents of new patients and patients with limited English proficiency were not included. Participants who completed at least one TriVox Health ADHD questionnaire between October 1, 2014 and December 31, 2015 were included in this analysis. Teacher responses were not programmed to trigger alerts and consequently were not considered in this analysis.

### 2.3. TriVox Health Platform

TriVox Health is a web-based platform that enables clinicians to administer online clinical questionnaires to parents and teachers to monitor patients remotely, between visits. Clinicians specify the interval for data collection, tailoring to a patient’s clinical status or medication regimen. For this study, questionnaires were assigned monthly. Respondents received a notification by either email or the TriVox Health mobile “app” to complete the surveys.

The TriVox Health ADHD questionnaire included the following surveys—current medication confirmation, a medication side effects inventory, the eighteen-item Vanderbilt ADHD Parent Rating Scale, the single-item modified Clinical Global Impression-Severity (CGI-S) scale, and the Clinical Global Impression-Improvement (CGI-I) scale. After parents and teachers completed questionnaires, clinicians received an email notification that new data are available to view. An “ALERT” label appeared if there was potentially actionable data, as defined by the trigger algorithms described below.

### 2.4. Interventions: Trigger Algorithm and Alert Resolution Process

**Figure 1:**
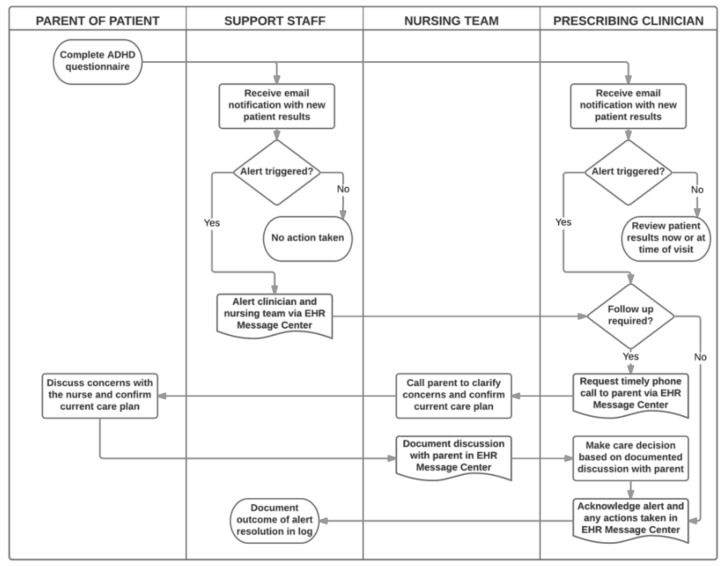
Alert resolution process.

We embedded the primary intervention, the trigger algorithm, into TriVox Health and created an alert resolution process as a secondary intervention. To develop the trigger algorithm, a multidisciplinary workgroup of ADHD experts reviewed the ADHD survey questions to identify parent responses on the current survey that would indicate a potentially actionable information warranting timely clinician review (Table 1). When parent responses met one or more of these criteria, the TriVox Health algorithm triggered an emailed alert notification to both the child’s ADHD clinician and to the TriVox Health staff. This initiated the alert resolution process (Figure 1), whereby support staff used the EHR message system to contact the Neurology nursing team, as well as the ADHD clinician. This communication method ensured that the care team saw the urgent patient information and documented it within the patient’s medical record. Clinicians chose whether to advise the nursing team to call parents, based on their clinical judgment and knowledge of the child’s history and baseline condition. At minimum, clinicians acknowledged receipt of the alert and documented actions taken to close the loop.

### 2.5. Outcome Measures

**Figure 2:**
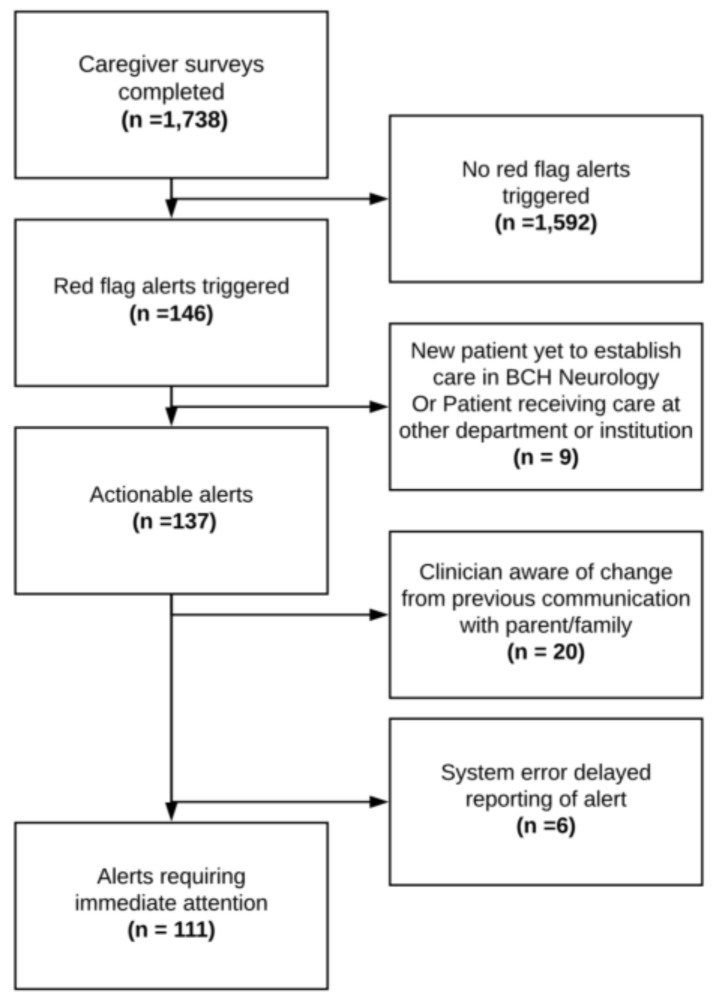
Actionable Alert Identification Tree.

**Figure 3:**
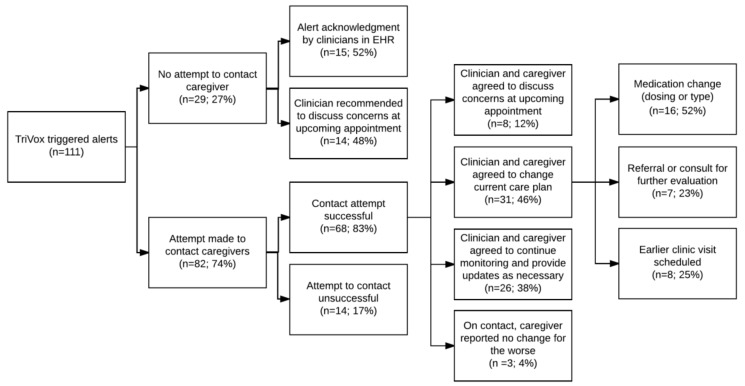
Alert Resolution Outcomes.

The primary outcome of interest was the action taken by clinicians as a result of the alert notification process, as documented in the medical record. Five investigators (AA, FO, JO, MC, and SG) reviewed and categorized clinician responses to triggered alerts. Two investigators independently reviewed and categorized each documented response. When the two categorizations differed, the entire group reviewed the case to reach consensus. First, clinician responses were classified by whether a caregiver was contacted and then by the subsequent care decisions—earlier outpatient clinic appointment scheduled, medication adjusted, referral for further evaluation made, or reassurance with no further actions provided. We excluded alerts in circumstances where clinicians would be unlikely to intervene ( Figure 2; Figure 3).

We also examined the relationship between the triggered alerts, preceding changes in patients’ ADHD severity score, and global functioning, as secondary outcomes. We divided participants into two groups—those who triggered an alert (alert group) and those who did not (non-alert group). ADHD severity was measured using the total score on the Vanderbilt, which asked parents to rate the frequency of each of the eighteen core DSM-IV criteria for ADHD [8,25,26]. Higher scores, with a maximum of 54, represent more severe ADHD symptoms. Global functioning was measured using the modified parent reported CGI-S scale, which asked parents to rate overall functioning and impairment from one (needs 24-h professional care) to seven (excellent functioning) [27,28,29].

To measure change in these scores, we compared parent ratings at the time of the triggered alert (Time 2) to the most recent ratings, completed within 90 days prior to the alert (Time 1). If patients generated more than one alert, we included the earliest alert that fulfilled these criteria. For patients who did not generate alerts, we measured changes in rating scale scores between the two earliest consecutive parent ADHD questionnaires, completed within 90 days (Time 1 and Time 2).

### 2.6. Process Measure

To assess timeliness of clinician responses to potential adverse events, we measured time to earliest documented follow-up. This was defined as the time in days, between the date the alert was triggered in TriVox to the date the clinical team documented their response in the EHR, including weekends and hospital holidays.

### 2.7. Balancing Measure

We measured the proportion of completed questionnaires, per clinician, that triggered an alert as a proxy for alert response burden.

### 2.8. Analysis

Patients were the unit of analysis. We conducted bivariable analyses to compare demographic characteristics, including race, ethnicity, sex, and insurance type, between the alert and non-alert patient groups. T-test analysis was used for age. Differences in Vanderbilt and CGI-S scores between patient groups were assessed by a regression analysis and adjusted for demographic variables that differed significantly between groups. A *p* value less than or equal to 0.05 was considered significant. Data were analyzed using STATA 13 (College Station, TX: StataCorp LP).

## 3. Results

Out of one hundred and thirteen ADHD clinicians, eighty-eight agreed to participate in TriVox Health. Between October 2014 and December 2015, at least one parent or guardian of one thousand and seventy-five children with ADHD, agreed to register in TriVox Health. During this timeframe, one thousand seven hundred and thirty-eight parent ADHD questionnaires were completed for five hundred and eighteen patients and their participating ADHD clinicians, which included 36% of attending physicians (30/83), 77% of nurse practitioners (7/9), 40% of residents (6/15), and 33% of fellows (2/6). Parent responses triggered at least one automated alert one hundred and forty-six ADHD questionnaires for ninety-eight patients and eighteen participating clinicians.

### 3.1. Patient Characteristics

The mean age of the 518 patients was 11 years. Patients in the alert group were on average 1.2 years younger than those in the non-alert group (95% CI: 0.5–1.9 [*p* < 0.001]). Patients who generated alerts were more likely to have public insurance, compared to those who did not generate alerts (*p* = 0.01). The groups did not differ significantly with respect to sex, race, and ethnicity (Table 2).

### 3.2. Primary Outcome

The algorithm uniquely identified one hundred and forty-six alerts (triggered by ninety-eight patients); one hundred and eleven (76%) alerts were considered actionable within a reasonable timeframe (Figure 2). Thirty-five alerts were excluded because patients were not currently receiving care within the Neurology Department (9 alerts), clinicians were made aware of the issue by another method (20s alert), or the system delayed alert reporting beyond an actionable timeframe (6 alerts). The median frequency was one alert per patient.

For eighty-two out of one hundred and eleven (74%) alerts, a nurse attempted to contact parents at the primary ADHD clinician’s request, reaching 83% of the families. When parents were reached, parents and clinicians agreed to change the care plan 46% of the time. Changes in the care plan included medication adjustments (52%), scheduling an earlier clinic visit (23%), and referral or consult for further evaluation (23%). Parents reported that they had not observed worsening in their child’s ADHD symptoms when contacted by the care team in only 4% (3/68) of cases (Figure 3).

### 3.3. Secondary Outcomes

Sixty-two out of ninety-eight patients (63%) who generated an alert (alert group) and two hundred and two (48%) patients who did not generate an alert (non-alert group), completed two consecutive ADHD questionnaires within 90 days. After adjusting for age and insurance status, alert group patients had higher Vanderbilt scores at Time 2 than the non-alert group, by 6.5 points on average, indicating a worse ADHD severity at Time 2 (95% CI: 3.9–9.0 [*p* < 0.001]). On average, the Vanderbilt scores in patients who triggered alerts increased by three points (SD: ±9.5) between Time 1 and Time 2, while the Vanderbilt scores in patients who did not trigger alerts decreased by two points (SD: ±7.4), indicating a worsened ADHD severity for the alert group and an improved ADHD severity for the non-alert group (Table 3). This difference in the magnitude of change of Vanderbilt scores between the two groups was 5.8 points higher, on average, in the alert group, when compared to the non-alert group, after adjusting for age and use of public insurance (95% CI: 3.5–8.1 [*p* < 0.001]).

After adjusting for age and insurance status, alert group patients had lower global functioning (CGI-S) scores at Time 2 than non-alert group patients, by an average of 0.3 points on the 1 to 9 point rating scale, indicating a worse global functioning at Time 2 for patients who triggered alerts (95% CI: 3.9–9.0 [*p* = 0.015]). On average, CGI-S scores alert group patients decreased by 0.2 units (SD: ±0.9) between Time 1 and Time 2, while the non-alert group’s CGI-S scores increased by 0.1 units (SD: ±0.8), indicating a worsened global functioning for the alert group and improved global functioning for the non-alert group (Table 3). This difference in the change in the CGI-S scores between the two groups was 0.3 points lower, on average, in the alert group, when compared to the non-alert group, after adjusting for age and use of public insurance (95% CI: −0.5–−0.1 [*p* = 0.015]).

### 3.4. Process Measure

The median time from alert to earliest documented clinician follow-up was two days, including weekends and hospital holidays (interquartile range (IQR) = 1–3 days).

### 3.5. Balancing Measure

Within the fourteen-month period, the median proportion of completed questionnaires, per clinician, that triggered an alert was 8% (IQR = 0–14%).

## 4. Discussion

Automated clinical alert algorithms have emerged as tools for error-reducing electronic health records, improving patient safety, and preventing potential adverse events. The remote electronic collection of parent ADHD rating scales between ambulatory visits can improve parent engagement and quality of care [8,9,10]. However, requiring clinicians to review all ADHD parent reports immediately to rule out acute worsening of symptoms could lead to data overload, alert fatigue, and delayed intervention. The ADHD trigger algorithms piloted in this study reduced the number of parent reports requiring immediate review to 8%, making it feasible for clinicians to follow-up between regularly scheduled visits to address urgent parent concerns that could have been missed. Our findings demonstrated that triggered alerts did not place undue burden on clinicians. Frequent and clinically insignificant alerts can desensitize clinicians, resulting in failure to respond and posing a risk to patient safety [12,13,14,15,30,31,32,33,34]. Triggered alerts in Computerized Physician Order Entry (CPOE) systems that lack clinical significance are often overridden by clinicians and fail to prompt clinical intervention [14,15,31,32]. Studies have shown that clinicians override up to 95% of automated alerts in the CPOE systems [13]. In the present study, clinicians provided written acknowledgement of all alerts in the EHR message system but did not take immediate action for 26% of alerts. Clinicians perceived 74% of alerts to be significant enough to prompt urgent follow-up with parents and nearly half (46%) of discussions with parents, initiated changes in the care plan, prior to the next scheduled outpatient appointment. These results suggest that clinicians were engaged in the process and actively intervened on most alerts, using their clinical knowledge of the patient to determine whether the triggering symptom should be addressed, urgently, or monitored until the next visit.

Alert algorithms within the CPOE systems often have high sensitivity but low specificity, leading to false positive alerts that contribute to high override rates and alert fatigue [32]. Two findings in our study suggest that the alert algorithms minimized false positive alerts. First, only 4% of triggered alerts were later ruled out when the parents did not confirm worsening in their child’s symptoms. Second, patients who generated alerts showed a significantly worsened ADHD severity and global functioning, when compared with patients who did not generate alerts (who showed improved ADHD severity and global functioning), suggesting that alerts captured patients with worsened ADHD severity and global functioning, within a ninety-day period.

Several issues limit the interpretability of our findings. First, this intervention was not randomized or blinded due to ethical concerns with collecting parent-reported information that may demonstrate worsening symptoms without intervening. Without a control group for comparison, it is unclear whether triggered alerts increased the likelihood of care interventions. Second, the burden of alerts on clinicians may have been minimal in part because the response rate was low; only 48% (518/1075) of registered patients completed at least one ADHD questionnaire. Third, although we observed a statistically significant difference in the short-term changes in the parent-reported Vanderbilt scores between groups, the clinical significance of this difference was not well studied, and the difference may be less sensitive in children with inattentive type ADHD. In addition, the CGI-S is yet to be validated for parent response. We did not use the Vanderbilt or the CGI-S scores as part of the trigger algorithm, because neither of the instruments were normed to yield a standardized score that enabled comparisons across individuals or groups. Finally, this study did not directly assess patient outcomes of the alert resolution process, nor was it designed to continuously monitor effects of the ADHD medication on heart rate and blood pressure [35].

Several factors contributed to the success of the trigger algorithms in this clinical setting but might have limited generalizability. Participation in TriVox Health was limited to parents with access to computers or mobile devices to complete online questionnaires between visits. This could present challenges in transferability to lower resource settings, though access to mobile devices has increasingly become equally distributed by race, ethnicity, and income [36]. Second, at the time of this study, TriVox Health was not yet designed to allow parents to initiate surveys on an on-demand basis, thus limiting the timeliness of parent reporting when their child experienced worsened symptoms or medication side-effects. However, scheduled monthly surveys still represents an improvement in how frequently children with ADHD are monitored with rating scales [37]. In addition, TriVox Health is an encrypted website that runs outside of the EHR, which makes it flexible and easy to access but presents additional challenges for integration and protection of data between electronic systems. The alert resolution process described in this study was developed to optimize the use of TriVox Health, within the context of this unique care environment. However, it relied upon sufficient resources, such as adequate support staff and nurses to ensure a timely follow-up, as well as an EHR with electronic messaging capability. This process could be modified to meet the resource availability of different care settings.

Importantly, our trigger algorithm has facilitated the integration of non-EHR based ADHD remote monitoring data into the clinical workflow, while minimizing the burden to clinicians. By significantly reducing the number of patient records requiring urgent review, the algorithm will allow clinicians to intervene efficiently between visits, for a small cohort of patients, while allocating the majority of time and resources toward preparing for ambulatory visits, including reviewing trends in online parent reports. The literature suggests that remote monitoring and other self-management tools empower patients and caregivers to be active participants in their care [38,39,40,41,42]. Our intervention takes patient-centered care a step further by encouraging clinicians to initiate communication with families between face-to-face visits when clinically necessary. Early intervention, as a result of this type of communication has been shown to improve patient satisfaction, patient outcomes, and decrease costs [41,43].

## 5. Conclusions

Clinicians were not inundated by alerts, perceived alerts to be clinically relevant, and frequently intervened on alerts by calling and engaging parents in medical decision-making, prior to their next appointment. Alert algorithms captured a group of patients who demonstrated significantly worsened symptoms, as measured by validated scales for ADHD severity. This novel application of an alert algorithm that filters electronic parent reports offers a significant value for clinical care of children with ADHD. Similar interventions have a strong potential to improve patient safety and quality for many other pediatric chronic conditions that require routine monitoring.

## Figures and Tables

**Table 1:** TriVox Health Attention-Deficit/Hyperactivity Disorder (ADHD) Trigger Algorithms.

Question Item	Survey Source	Response Triggering Alert
Increased hostility or aggression	Symptoms and side effects severity rating	“Increased hostility and aggression” (mild, moderate, or severe) AND “change for the worse” ^1,2^
At risk of hurting self or others	Symptoms and side effects severity rating	“Expresses thoughts of hurting self or others (mild, moderate, or severe)” AND “change for the worse” ^1,2^
Clinical improvement since start of treatment or start of the most recent change in treatment	CGI-I	Clinical improvement rated as “much worse” OR “very much worse” ^2^

^1^ The expert workgroup, with extensive feedback from front-line clinicians, determined that if a symptom was endorsed at the triggering severity level but was deemed to be stable (i.e., no change for the worse), the response would not trigger an alert. ^2^ “Change for the worse” is based on parents’ assessment of the child’s current vs. previous (or “usual”) state.

**Table 2:** Patient Characteristics.

		Alert Group (N = 98)	Non-Alert Group (N = 420)		
	N	mean (SD)	mean (SD)	Δ (95% CI)	*p*
Age (years)	518	9.85 (3.21)	11.09 (3.24)	1.2 (0.5, 1.9)	<0.001 *
	N	N (%)	N (%)	OR (95% CI)	*p*
Sex				0.6 (0.34, 1.06)	0.08
Female	126	17 (17.4%)	109 (26.0%)		
Male	392	81 (82.7%)	311 (74.1%)		
Race (56 missing)				1.32 (0.60, 2.91)	0.49
White	406	72 (90.0%)	334 (87.2%)		
Non-White	57	8 (10.0%)	49 (12.8%)		
Ethnicity (114 missing)				1.21 (0.48, 3.08)	0.69
Hispanic	30	6 (8.6%)	24 (7.2%)		
Non-Hispanic	374	64 (91.4%)	310 (9.8%)		
Insurance (7 missing)				1.79 (1.14, 2.81)	0.01 *
Any public insurance	164	42 (42.9%)	122 (29.6%)		
Private	347	56 (57.1%)	291 (70.5%)		

* A *p* value less than or equal 0.05 was considered significant.

**Table 3:** Ninety-day change in mean Vanderbilt and Clinical Global Impression-Severity (CGI-S) scores for patients who generated an alert vs. those who did not.

**Vanderbilt Scores (Range: 0 [Least Severe] to 54 [Most Severe])**				
	Alert Group (N= 62)	Non-Alert Group (N = 202)		
	mean	mean	Δ (95% CI)	*p* *
Time 1 **	24.8	22.1		
Time 2	28.2	20.2		
Diff	3.4 (SD: ±9.5)	−1.9 (SD: ±7.4)	5.8 (3.5, 8.1) ***	0.001
**CGI-S scores (Range: 0 [Most Impaired] to 9 [Least Impaired])**				
	Alert group (N = 61)	Non-Alert group (N = 202)		
	mean	mean	Δ (95% CI)	*p* *
Time 1 **	4.8	5.3		
Time 2	4.6	5.4		
Diff	−0.2 (SD: ±0.9)	0.1 (SD: ±0.8)	−0.3 (−0.5, −0.1) ***	0.015

* A *p* value less than or equal to 0.05 was considered significant. ** No statistical difference between both groups at time 1. *** After controlling for age and insurance.
